# Sculptors, Architects, and Painters Conceive of Depicted Spaces Differently

**DOI:** 10.1111/cogs.12510

**Published:** 2017-06-27

**Authors:** Claudia Cialone, Thora Tenbrink, Hugo J. Spiers

**Affiliations:** ^1^ Centre of Excellence for the Dynamics of Language College of Medicine, Biology and the Environment School of Psychology Australian National University; ^2^ School of Linguistics and English Language Bangor University; ^3^ Department of Experimental Psychology Division of Psychology and Language Sciences Institute of Behavioural Neuroscience University College London

**Keywords:** Linguistics, Psychology, Discourse, Culture, Semantics, Concepts

## Abstract

Sculptors, architects, and painters are three professional groups that require a comprehensive understanding of how to manipulate spatial structures. While it has been speculated that they may differ in the way they conceive of space due to the different professional demands, this has not been empirically tested. To achieve this, we asked architects, painters, sculptors, and a control group questions about spatially complex pictures. Verbalizations elicited were examined using cognitive discourse analysis. We found significant differences between each group. Only painters shifted consistently between 2D and 3D concepts, architects were concerned with paths and spatial physical boundedness, and sculptors produced responses that fell between architects and painters. All three differed from controls, whose verbalizations were generally less elaborate and detailed. Thus, for the case of sculptors, architects, and painters, profession appears to relate to a different spatial conceptualization manifested through a systematically contrasting way of talking about space.

## Abstract 2

video abstract: https://youtu.be/w7dsVjWRitI

## 1 Introduction

More than 70 years ago, Whorf (1941) formulated an intuition that has shaped research across various disciplines ever since: Language is part of human nature, and thus intimately related to human thought. The exact nature of this relationship has been a matter of extensive debate, both concerning the extent to which language influences thought (moderate linguistic relativity, i.e., correlation—or strong linguistic determinism?) and the direction of causality—does the structure of language shape the structure of thought, or vice versa (Levinson, Kita, Haun, & Rasch, 2002; Li & Gleitman, 2002)? Central insights in this area were gained from cross‐linguistic comparative studies. Linguistic structure and language use differ across cultures, as does cognition—both are related in intricate ways (Levinson, 2003), and they draw on inherent cognitive and biological biases (Haun, Rapold, Call, Janzen, & Levinson, 2006). Many studies promote the idea that language directly shapes thought, both generally (Boroditsky, 2011) and situationally (Lupyan, 2012), in that specific lexical elements and grammatical structures of a language constrain thought processes when speaking (“thinking for speaking”: Lucy, 1992; Norcliffe, Konopka, Brown, & Levinson, 2015; Slobin, 1996, 2000). However, language structures also appear to adapt to the sociocultural environment in which they are learned and used (Lupyan & Dale, 2010), and they develop through the functions of embodied use and entrenchment in cultural necessities, both ontogenetically and phylogenetically (Evans, 2014).

The Whorfian question continues to be a matter of widespread interest with a rich diversity of perspectives. However, it is less widely known that Whorf's intuitions were not originally inspired by cultural or linguistic differences (though substantiated by those), but primarily by his experience as a fire insurance executive (Whorf, 1941). His argument begins by illustrating cognitive effects of simple words like “empty,” where the use of the term “empty gasoline drums” can lead to careless behavior. Whorf argued further that language crucially reflects the linguistic habits of a group, as well as their way of thinking about the world. While the direction of causality is not central to this original line of argument, the deeper insight that has been overlooked in research so far is this: If culture is intricately related to thought and language, then this should also be true for *profession*. A person's individual background necessarily shapes her thinking; this includes both cultural and professional aspects, at least after a number of years. Accordingly, professional experience, intended as the acquisition and practice of skills over a relatively long period of time, should affect a person's way of speaking about states and relations in the world.

This insight leads to a range of issues that have only partially been addressed empirically so far. Relevant research deals, for instance, with the features of expert language, with extensive studies centering on notions of register (Bhatia, 1993; Schleppegrell, 2004) and English (or Languages, in general) for specific purposes (Hutchinson & Waters, 1987). Some research has addressed the ways in which experts adapt their communication to non‐experts (Bromme, Rambow, & Nückles, 2001; Isaacs & Clark, 1987). While these strands of research primarily address language rather than cognition, some studies also indicate that verbalizations of cognition reflect expertise in systematic ways (Tenbrink, Bergmann, & Konieczny, 2011; Van Gog, Paas, & Van Merriënboer, 2005), and that professional background can result in substantial neurocognitive diversity (e.g., in taxi drivers and bus drivers; Maguire, Woollett, & Spiers, 2006). Expertise also affects cognition in terms of how visual information is perceived and reasoned about (Chase & Simon, 1973; Peebles, 2013; Shipley, Tikoff, Ormand, & Manduca, 2013). In the area of mental imagery, it was found that domain‐related visual experience affects the ability to infer relevant spatial relationships from the presented textual information (Noordzij, Zuidhoek, & Postma, 2006).

Common to previous research around expert language and cognition is the focus on variation along a scale of expertise. Expert insights have been investigated precisely in areas for which they are relevant. What appears to be underlying this common trait is the unspoken assumption that professional expertise only affects cognition where expertise is at stake directly—that is, without altering human thought in a more fundamental sense. Based on the above‐mentioned body of evidence showing the profound impact of cultural background on language and thought, we question this assumption and ask if professional background affects human cognition in fundamental ways. Intuitively this seems straightforward enough: If cultural activities shape our thought, so would, more specifically, professional background. However, this aspect appears to have evaded systematic study entirely so far.

A frequent domain for addressing the relation between language and thought is space, due to its ubiquitous relevance and manifold effects on human development (Newcombe & Huttenlocher, 2003; Waller & Nadel, 2013). The spatial domain serves as a focal area in which the intricate intertwinement between language, culture, and cognition is played out. For instance, spatial mental imagery has been extensively investigated through language (Huttenlocher, 1968), both in terms of the imagery created while reading (Langston, Kramer, & Glenberg, 1998; Zwaan & Radvansky, 1998), and in terms of the spatial features that emerge in verbalization (Levelt, 1996; Taylor & Tversky, 1992), and with differential effects according to cultural background (Mainwaring, Tversky, Ohgishi, & Schiano, 2003). Here, we address the relation between spatial language and cognition by focusing on profession as a possible influential variable. Specifically, we focus on professions in which space plays a distinct role, but in different ways: painters, sculptors, and architects. While our study cannot determine any direct causal effects of professional training on spatial concepts and language use, we aim to identify patterns of verbalizations in the description of space in images related to profession.

## 2 Spatial professions, cognition, and language

Painters, sculptors, and architects all must develop a highly attuned understanding of the arrangement of structure in space to succeed in their professions. Le Corbusier (1945) argued that these three groups of trained individuals in particular are equipped with a “feeling of space,” practitioners of the spatial science “*par excellence*.” Intuitions like this are scattered throughout the literature (see e.g., Levinson, 2001). Philosophical and esthetic reflections around painting, sculpture, and architecture frequently deal with the theme of space, such as perception and depiction of space in paintings and photographs (Hopkins, 2001, 2003, 2004; Langer, 1953; Sayre, 1989; Schier, 1986; Vance, 1995; Wollheim, 1980, 1987). Those training in painting, sculpture, and architecture need an education in how to consider, manipulate, and create spaces, whether two‐ or three‐dimensional (Krauss, 1979, 1981; Lefebvre, 1991; Schmarsow, 1893). Thus, it would seem likely that these professionals also possess a different way to describe and talk about space as compared to people from other professions, implying a more extensive, nuanced, and detailed use of spatial language.

Surprisingly little research has addressed the spatial language use of architects, painters, and sculptors. Beyond the use of general‐purpose terms such as spatial prepositions or action verbs, professional training should include specific terminology (e.g., terms used in the casting process for sculpture, or for forming an initial sketch or a plan). Since expert terminology refers to expert concepts, the specific professional language might more fundamentally foster distinct conceptualizations of space. For example, architects frequently reason with two‐ and three‐dimensional representations of space (Al‐Sayed, Dalton, & Hölscher, 2010; Dalton, Höelscher, & Spiers, 2010; Hölscher & Dalton, 2008), which can be used, for example, to help “design the limits that give the impression of space” (Souto de Moura, 2014).^1^ In addition, architects are required to perform a substantial amount of spatial transformation and perspective taking (Brösamle & Hölscher, 2007). In contrast to architects, painters translate aspects of the 3D (real) world to a 2D surface. Since painting does not directly entail modifying the real world, this allows attention to be focused directly on the 2D visual configuration. Like architects, sculptors deal with 3D space (Hopkins, 2003, 2004), but with respect to a scale of space that is more similar to the painters. In contrast to architects, for both sculptors and painters, there is no formal requirement for the constructed form to be functional for human use. In sum, there are good reasons to expect that architects, painters, and sculptors differ in the way they consider and describe space. However, to date, there has been little empirical exploration of this idea.

A traditional method for investigating human thought processes is through the analysis of verbalizations (Ericsson & Simon, 1984), with many applications in the spatial domain (e.g., Gugerty & Rodes, 2007; Pick, Heinrichs, Montello, Smith, & Sullivan, 1995; Suwa & Tversky, 1997; Spiers & Maguire, 2006, 2008). Extending this tradition, cognitive discourse analysis (CODA; Tenbrink, 2015) targets not only *what* is said but more specifically *how* it is said. Drawing on insights demonstrating the significance of specific linguistic patterns (e.g., Evans & Green, 2006; Halliday & Matthiessen, 2014; Talmy, 2000), CODA highlights specific aspects of thought underlying linguistic choices, beyond the explicitly formulated content that speakers are consciously aware of. In the domain of space, CODA has been applied to address route planning at different scales (Hölscher, Tenbrink, & Wiener, 2011; Tenbrink & Seifert, 2011; Tenbrink & Wiener, 2009; Tenbrink, Bergmann, et al., 2011), as well as various kinds of spatial conceptualizations (Tenbrink, Coventry, & Andonova, 2011; Tenbrink & Salwiczek, 2016).

In this study, we used CODA to analyze data collected in a task involving the description of images depicting complex spatial environments. We aimed to determine if architects, painters, and sculptors (henceforth “spatial professionals”) differ between each other in their conception of space as reflected in the use of spatial language in a task not requiring their specific expertise. Furthermore, we asked if spatial professionals differ from people with non‐spatial professions (henceforth “controls”) in this regard. We predicted that the spatial professionals’ experience should manifest itself through a different way of describing space as compared to controls. Architects and sculptors should be particularly concerned with the real‐world aspects of the environments depicted in the images, including their materiality or haptic features (e.g., solidity, touch, materials used in them), or 3D structure of the spaces, while painters should focus more on other aspects related to space of the images as such.

## 3 Methodology

This research was approved by the ethics committee in the Division of Psychology and Language Sciences, University College London.

### 3.1 Participants

All participants in the spatial professional groups (architects, painters, sculptors) had at least 8 years of experience (including training and professional work) in only one of these three professions. The number of years was decided as a suitably long amount of time to acquire consistent experience within one discipline. Participants were recruited across a number of artist or architect studios in and around London, randomly chosen to avoid drawing on a specific subgroup within this population. Controls did not have any background related to the three spatial professions, nor did they engage consistently in day‐to‐day art activities or any other professional occupation requiring particular spatial abilities and skills or a more focused spatial awareness, such as geospatial science, spatial cognition, engineering, geography, or related disciplines (cf. Hegarty, Crookes, Dara‐Abrams, & Shipley, 2010). They were recruited as part of the “Psychology and language science” department and included IT managers, administration officers, cognitive psychologists, and some PhD students at UCL with at least 6 years of experience in their profession, who were in the same age range as the spatial professionals. The language used throughout this study was English; all participants were English native speakers. Sixty‐four subjects were included in this study (see Table 1 for details).

**Table 1:** Cross‐group average age and years of professional experience

Group of Profession	Number of Female/Male Participants	Mean Age (SD), Range	Mean Years of Professional Experience (SD), Range
Architects	8/8	37.1 (11.7), 26–66^*s,p*^	16.2 (8.2), 8–32^*s,p*^
Sculptors	8/8	50.9 (9), 31–63^*a,c*^	27.9 (9.4),15–46^*a,c*^
Painters	6/10	50.4 (10.4), 36–68^*a,c*^	28.9 (9.7), 22–57^*a,c*^
Controls	8/8	39.6 (9.3), 29–61^*s,p*^	17.7 (11.4), 6–49^*s,p*^

Notes.

Post hoc Sidak results are indicated as follows: *a* (*architects*) = sign. different to architects *p* < .05, *s* (sculptors) = sign. different to sculptors *p* < .05, *p* (*painters*) = sign. different to painters *p* < .05, *c* (*controls*) = sign. different to controls *p* < .05.

While we attempted to match groups for age and experience, we found that following recruitment significant differences across groups for age (*F*(3, 60) = 7.94, *p* < .001) and years of experience (*F*(3, 60) = 7.43, *p* < .001) emerged. Architects and controls tended to be younger and have fewer years of professional experience than painters and sculptors (see Table 1). Using age and years of experience as co‐variates in our analysis below, we ascertained that this variance in background demographics did not explain our results. Follow‐up GLM analyses showed that introducing the covariates age and years of experience into the model did not mitigate the significant effects observed in our main analysis (Appendix S4).

### 3.2 Materials

**Figure 1:**
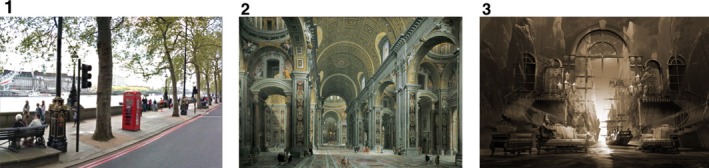
Pictures presented to participants in the task. The sources are as follows: (1) a Google street view shot; (2) a copy of a painting by Parini; (3) a copy of a computer‐generated creation by the graphic artist George Grie. http://neosurrealismart.com/modern-art-prints/?artworks/mindscape-or-virtual-reality-dreamscape.html

Six horizontally oriented, A4‐sized laminated pictures, each representing a different environment, were used as stimuli. Only data from the first three were analyzed (see Fig. 1), namely: (1) a Google street view shot of an urban outdoor environment; (2) a painting of the interior of St. Peter's cathedral; (3) a computer‐generated virtual composition of superimposed indoor/outdoor environments. The remaining three pictures were (4) a Google street view image of an English country‐side view; (5) a photograph of a contemporary indoor environment—a university hall; (6) a drawing of a surreal urban environment. These three pictures were presented after the first three used in our analysis. We excluded them purely to constrain our analysis to manageable time limits. All the pictures were chosen to provide engaging examples of visual spatial scenes that varied in terms of real/surreal, outdoor/indoor, contemporary/historical, photograph/painting/computer‐generated, lighting condition (bright, medium, gloomy), and geometry and perspective (frontal asymmetric, lateral, frontal symmetric). They were also chosen for their complex layout, and for the absence of a salient person or group of people. In this way we intended to elicit predominantly descriptions of the spaces depicted.

Verbal descriptions were audio‐recorded using Audacity software version 2.0.5. The tool F5 version 2.1 was used to facilitate transcription, Microsoft Excel version 14 for calculations and automatic word search, and IBM SPSS version 21 to implement and evaluate statistical models (Field, 2009).

### 3.3 Procedure

Each participant was interviewed individually by the same experimenter, at a site of their choice to ensure familiarity and comfort, adequate to the nature of this study with its focus on the participants’ individual background. The experimenter made sure that illumination was adequate for perception of the pictures (natural light or electric source).

Before starting the interviews, participants read and signed a consent form and provided their demographics. With the start of recording, the experimenter read an instruction script to the participants, who were then allowed to ask clarification questions (see Appendix S1). Next, as a warm‐up task, participants completed the PANNS (see Appendix S2) abstract thinking test (Kay, Flszbein, & Opfer, 1987), which involves defining similarity between four pairs of items and explaining the meaning of four English proverbs. Participants were informed that the test would be used “to test their abstract thinking abilities.” Apart from the warming‐up function there was no relation between the PANNS test and the current study.

Next, participants were shown the visual stimuli in the sequential order shown in Fig. 1. They were asked the same three questions for each picture:

1. “Could you please describe the environment that you see in this picture?”
2. “How would you explore the space in this image, where would you go?”
3. “If you were given the chance, how would you change the environment in this image?”

These questions were chosen to address three aspects of interest: “description”; “exploration,” and “transformation” of the spaces represented. This allows for a gradual increase of engagement with the images, starting from a static view of the scene followed by a more dynamic conceptual tour, culminating in the idea of affecting and altering the scene. Given that this last question required a more imaginative approach, we presented it as an opportunity given to the participants, namely to transform the environments depicted.

After all images were presented, participants in the spatial professional groups were furthermore asked (question 4): “What is ‘space’ for you?” This question was not posed to the control group.

Participants were free to answer the questions in any way they chose, without temporal restrictions. Interviews typically lasted for more than half an hour for the spatial professionals (PANNS, 6 images, and question 4), with a mean of 28:44 min for architects (range: 11:48 min, 59:44 max), 36:31 for painters (17:02 min, 60:04 max), and 34:30 for sculptors (15:51 min, 60:08 max). The average time for the controls (PANNS and 3 images) was 10:22 min (6:20 min, 22:49 max). This depended on the degree of verbosity in participants, which we account for in our analysis by calculating relative frequencies in the analyses below.

Apart from asking the questions, the experimenter remained passive during the interviews, unless the following cases occurred:

- the participant did not hear the question properly or signaled non‐understanding, and asked for a repetition, in which case the same question was repeated again;
- the participant signaled non‐understanding of the meaning of a question, in which case the experimenter would reply “whatever you think the question means”;
- the participant referred to or indicated a point in the image using a deictic pronoun (e.g., *there, that*). In this case, to ensure optimal transparency and clarity, the experimenter would ask what was referred to. This was done subtly to avoid affecting the natural discourse flow, namely by simply asking “where” or “what,” depending on whether the deictic term indicated a location (e.g., here/there) or an object or other specific phenomenon (e.g., this/that).

## 4 Analysis

The analysis of the collected language data was based on the techniques of CODA (Tenbrink, 2015). CODA involves the following steps to be outlined briefly in the following: *transcription*,* segmentation*,* annotation*, and checking for *intercoder reliability*. The results gathered on this basis are then analyzed both qualitatively and quantitatively.

*Transcription* was done on the level of content, disregarding hesitation markers, false starts, mispronunciations, and the like, since these were not targeted in the present analysis. Everything the participants said was transcribed conscientiously.

*Segmentation* of the verbal data was done for practical purposes and to enable quantification of relevant features identified in a segment. Following Suwa and Tversky (1997), a segment was defined as “one coherent statement about a single item/space/topic.” In practice, syntactic, prosodic, and semantic aspects were taken into account to identify a coherent segment for the purposes of the present analysis. Examples are “I won't change it” or “but when you put objects in it or put walls in it, you start to contain it.”

*Annotation* was based on operationalized definitions as follows. Definitions either related to the whole picture (I. below), to a specific part of it (II. below), or to a general conception of space (III., this refers to question 4). The Appendix gives specific operationalizations for each category along with examples; here is a summary.

I Whole picture
  - Category 1: Linguistic items representing flat (one and two‐dimensional Euclidean) geometry; focus on the two‐dimensional geometrical shape of the entities represented in the images.
  - Category 2: Materiality. Reference to the material and/or haptic features of the spaces depicted.
  - Category 3: Task clarification request. Content inspection revealed that some participants needed clarification before responding to some questions. Since this might signal some kind of cognitive mismatch, this was annotated systematically so as to identify any patterns.
  - Category 4: Exploration of the “spaces” in the images. Reference to imagined “exploration” (involving any action or motion) performed within the depicted space as if it was in the real world.
  - Category 5: Exploration of the “image” as such. Reference to visual (rather than physical) engagement with the depicted space when “exploring” it.
  - Category 6: Transformation of the “spaces” in the images. Reference to imagined three‐dimensional (physical) transformation of the depicted space.
  - Category 7: Transformation of the “images.” Reference to visual (rather than physical) transformation of the images.
II A specific part of the picture Inspection of the data showed that spatial professionals regularly referred to one central part of the picture, here defined as the “furthest point” in the 2D representation of 3D space. While this part of the picture could have been referred to in many different ways, the most frequent were these:
  - Category 8: Reference to furthest point as “back.”
  - Category 9: Reference to furthest point as “end.”
III General conception of space
  - Category 10: Mental representation of space as a bordered and enclosed physical reality. With respect to question 4, annotation captured whether space was referred to as a delimited, physically defined and contained reality, with perceivable boundaries. Following relevant literature in this area (Bateman, Hois, Ross, & Tenbrink, 2010; Talmy, 2000), this category includes mention of size/dimension measures, perceivable or physical borders, defined areas with a 3D structure (e.g., volumes), 2D defined surfaces, and shape.

The definition of conceptual‐linguistic categories was guided, on the one hand, by our predictions outlined above and, on the other hand, on a content‐based analysis and understanding of the interviews (Krippendorff, 2004). A first overall (human) read of all of the verbalizations and a following (automatic) scan of the datasets through Excel spreadsheets led to the identification of systematically occurring language indicators corresponding to central cognitive spatial elements known from the literature (e.g., Bateman et al., 2010; Bennett, 2006; Bennett & Agarwal, 2007; Herskovits, 1987; Jackendoff, 1983; Landau, 1991; Talmy, 2000, 2005). The categories relating to exploration and transformation of “space” in the picture were specifically informed by literature related to embodied cognition, where mental imagery is recognized as leading to a “*re‐enactment* of specific exploratory perceptual behavior that would be appropriate for exploring the imagined object as if it were actually present” (Barsalou, 1999; Holsanova, 2006, 2008). In contrast, a more analytical transformation and exploration of the “image” as such seems to be a more common pattern of the picture‐expert eye (Arnheim, 1960).

Annotation was done segment by segment, counting the number of occurrences of each of the linguistic indicators representing a conceptual category. While these indicators were associated with specific questions (e.g., descriptive linguistic indicators mainly pertained to question 1, exploration verbs of action and motion to question 2, verbs of action indicating transformation to question 3 etc.), they were still coded throughout the responses to questions 1–3 to allow for the dynamics of free language production. Accordingly, relative frequencies were calculated in relation to the overall number of words produced by each participant in questions 1–3.

*Intercoder reliability* was assessed as follows. Following training, a second person, who was blind to the goals of the study and did not know anything about the identity or the profession of the participants, independently scored a subset of the data (approx. 20% of all data, randomly selected across all conditions and groups but manually making sure that the verbalizations selected did not explicitly give away the identity of the participants). Krippendorff's alpha (Krippendorff, 2004) was computed separately for each category (see Appendix S3: 1 and 2 for details), using the SPSS macro described in Hayes and Krippendorff (2007). One calculation was carried out for the whole dataset except question 4 (not given to the controls), and another to calculate the levels of agreement for each scored subcategory of “space as a bordered and enclosed physical reality” in all conditions (only question 4). Results in both analyses reached satisfactory agreement with scores between 0.70 and 1.

A chi‐squared test was used to determine significant patterns in the distribution of participants, using specific types of language indicators in categories 3, 8, 9, and 10. Separate two‐tailed *t* tests were run, where necessary, to compare the means of relative frequency distribution between spatial professionals and controls. To account for inhomogeneity between the two samples (16 controls vs. 48 professionals), a Welch's *t* test was calculated (which provides non‐integer degrees of freedom). Because the relative count data was skewed toward zero, we applied a log(*x* + 1) transform to the data.

Further, a generalized linear mixed model (GLMM) analysis, with Šidak correction, was chosen for categories 1, 2, 4, 5, 6, and 7, 8, 9 to account for non‐normal distribution of verbal data and to cater for multilevel sampling. Due to differences in verbosity, the data were not normally distributed but were normalized through the link function in GLMM.

## 5 Results

The three spatial professional groups differed in their use of language both between each other and in comparison to controls. Painters’ language was characterized by the use of “back” when referring to the “furthest point” of a picture, by a higher need for clarification, and by shifts between 2D and 3D conceptions of space. Architects’ language was characterized by the use of “end” rather than “back” for the “furthest point” of a picture, by a focus on materiality, by an imagined exploration of the spaces depicted as real‐world (and so 3D) environments rather than 2D images as such, and by an understanding of “space” in terms of physical borders. Sculptors’ language was characterized by a combination of these features, with indicators often falling between those of architects and painters. Table 2 exemplifies the use of the main language indicators and units (underlined here for clarity, within a discourse context) across the four questions. In the following, we first compare the proportions of participants in each group who used certain language indicators and categories at all, and then turn to the relative frequencies of language indicators.

**Table 2:** Excerpts from participants’ reports

Question	Controls	Architects	Sculptors	Painters
Could you please describe the environment that you see in this picture?	*… a building façade or interior possibly superimposed on an image of the sea and the ship, and rocks, and it is quite dark, apart from the light* *in the center*	*… a body of water going down between huge cliffs disappearing into some bits … a lot of light toward the* *end*	*… it starts at the top as a mixture of architectural wall and a landscape … a chasm* *at the end* *of which it seems there is the setting sun*	… *there is a ship in the middle coming through a distorted window, a high arched window with what looked like mountains* *at the back*
How would you explore the space in this image; where would you go?	*I just like* *to explore* *and see what's there, obviously I can see what's here but behind the arches and behind that pillar…* *I'd just walk around and see* *all the hidden statues … and obviously* *looking up* *at the ceiling as well … so that's what I would do*.	*I would* *touch materials* *around, it is a very … the* *cold stone* *seems inviting* *to touch. I would want to go to* *the side‐walls* *and touch* *them*	*I always like* *to get up close to the surfaces* *of things … sort of see* *how the floors are laid and look at* *the panels on the pillars and then* *the copper* *ceilings and* *see how things were made…*	*I'd probably* *walk down* … *towards the crossing … maybe back into the nave here … now just* *looking at it as a two‐dimensional abstract image … my eye goes straight* *to this on the right … all* *these lines take my eye down* *… there's a bit of a yellow colour* …
If you were given the chance, how would you change the environment in this image?	*I suppose* *you could have a cycle lane* * … so that people can cycle along this street … hum … trees,* *more trees* *is always nice …*	*… to take away that barrier* *to the river, it be quite nice to be able to walk along the edge and feel you were directly over the water … so having a solid balustrade puts a bit of a barrier between you and the river …* *breaking about barriers* *between you and the pavement, and the pavement and the river …*	*there are a lot of objects already there …* *if I were to make a sculpture* *somewhere along this area **I*** *would remove* *the telephone box, which is extremely red as an object, a very powerful object … I* *would turn the sound on … plenty of seats for people to sit* *on …*	*I would want to* *straighten up the diagonal* *of the road and make it* *more flat* *to the bottom edge* *,* *flatten* *it down, I'd want to bring it down …* *less of an angle …*
What is “space” for you?	n/a	*I'm really interested in how* *buildings frame space* *, how* *walls frame space* *… space on its own is kind of meaningless … but when you put objects in it or* *put walls in it* *, you start to* *contain* *it … what we don't realize often is that what we're doing is* *framing space*.	… *that's the world that I think about a lot, that inspires my work … but also space in* *space travel* *and looking at space … and I like* *astronauts* *and those sort of things … It is the* *gap between solid objects* *but then* *solid objects are space* *themselves. Space is also* *where you live* *or your* *studios* *…*	*Space is sort of where the time comes from a long way away. Things from a long way away reach you at this kind of … in a sort of … light, from things millions, billions, trillions of years ago come or even that, even from the sun 9 min ago …*

### 5.1 Between‐group comparisons in the proportions of participants using specific linguistic or conceptual categories

**Figure 2:**
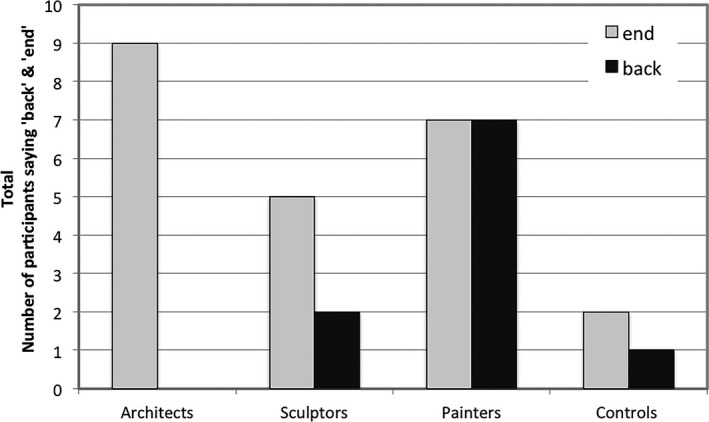
Number of participants using “back” or “end.”

**Figure 3:**
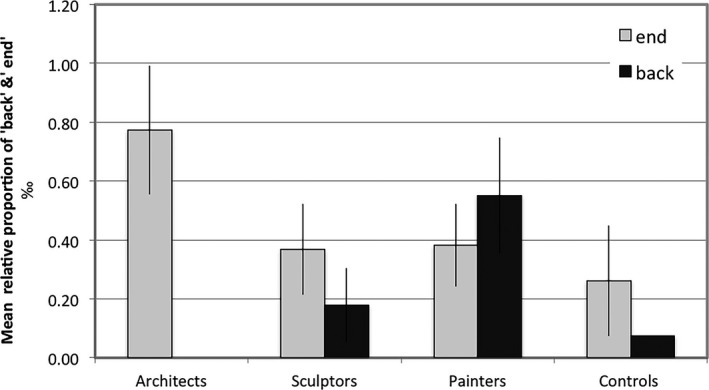
Relative mean proportions (per thousand) and standard errors for the use of “back” and “end.”

As shown in Fig. 2, a significantly larger proportion of painters referred to the furthest point of the pictures as “back” than architects (χ² = 8.96, *p* < .05), controls (χ² = 6.00, *p* < .05), and sculptors (χ² = 3.86, *p* < .05). Also, a significantly larger proportion of architects referred to the same region as “end” than controls (χ² = 6.79, *p* < .05). Painters also differed from controls in the use of “end” (χ² = 3.87, *p* < .05). Fig. 3 provides a visual inspection of relative frequency distribution for the use of “back” and “end.” Statistical analysis on this basis yielded no further results: A set of (two‐tailed) *t* tests comparing spatial professionals and controls yield only non‐significant tendencies for the use of “end” (*t* = −1.16, df = 24.54, *p* = .258). For “back,” *t* tests were not conducted given that only one control used it twice. This indicates that the previously found effects are primarily due to individual differences related to profession, rather than indicating frequency differences across individuals. However, a GLMM cross‐group analysis for the use of “back” approached significance (*F*(3, 120) = 2.452, *p* = .069), mainly driven by painters versus architects (*t* = 2.49, df = 120, *p* = .084). Controls*,* who rarely used either “end” or “back,” instead used other common spatial terms to refer to the same location, such as “center” or “bottom,” or the deictic term “there.”

**Figure 4:**
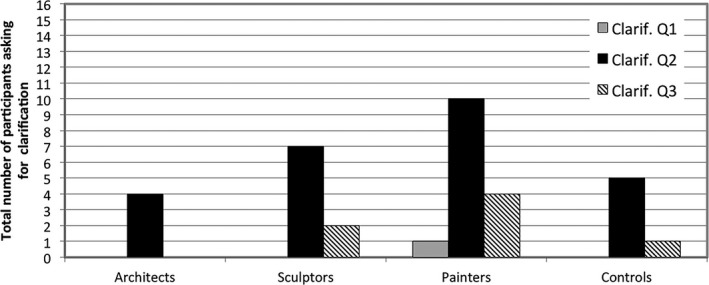
Number of participants asking for clarification.

Next, we examined the use of clarification requests to each of the three questions asked about the pictures. Significant differences were found only for question 2: “How would you explore the space in this image, where would you go?” Painters were more likely to ask for clarification of this question than architects (χ² = 4.57, *p* < .05, Fig. 4). The difference between painters and controls showed a trend toward significance (χ² = 3.14, *p* = .07), and the proportion of sculptors who requested for a clarification was numerically between painters and architects (without significant differences).

**Figure 5:**
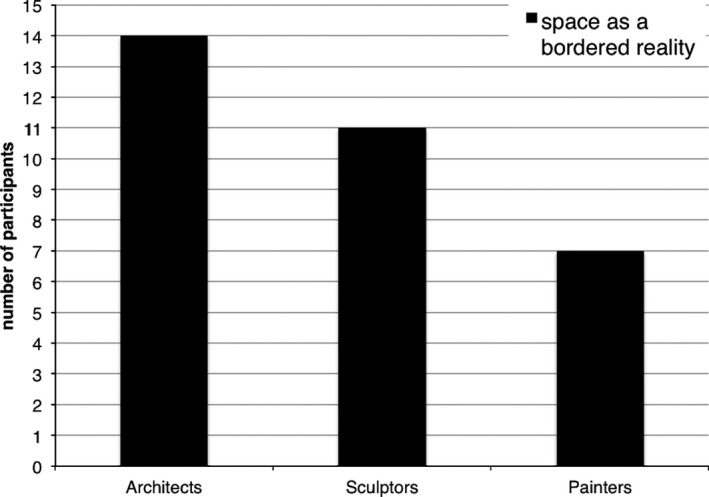
Number of spatial professionals defining space as a bordered physical reality in answer to question 4.

Finally, we examined the proportion of spatial professionals who described space as a bordered physical reality in response to question 4 (which was not posed to controls). Architects were most likely to do so, followed by sculptors and then painters. Architects were significantly more likely than painters to describe space as a bordered physical reality (χ² = 6.79, *p* < .005), (Fig. 5). A more fine‐grained analysis of question 4 will follow in the next session.

### 5.2 Between‐group comparisons of relative frequencies for specific linguistic or conceptual categories

**Figure 6:**
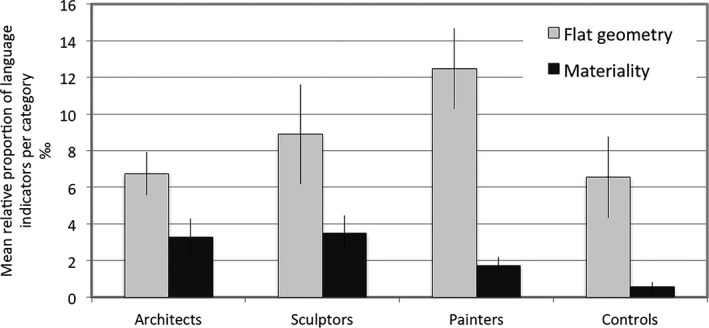
Relative mean proportions (per thousand) and standard errors for the use of linguistic indicators referring to flat geometry and materiality.

Fig. 6 highlights the different patterns of focus on materiality as compared to flat geometry. *t* tests conducted between spatial professionals and controls revealed a significant difference in the relative frequency of use of linguistic markers for the materiality of the depicted spaces (*t* = −4.11, df = 61.80, *p* < .0001). These results were then corroborated by GLMM analysis showing a significant difference across groups for this category (*F*(3, 120) = 3.59, *p* < .05). Between‐group comparisons revealed that sculptors were significantly more likely to use terms of materiality than controls (*t* = 2.84, df = 120, *p* < .05), and so were architects (*t* = 2.63, df = 120, *p* < .05). Painters did not differ from controls, as both groups used only few materiality indicators. There were no significant differences between the three spatial professional groups.

As for flat geometry indicators, an independent sample (two‐tailed) *t* test did not reveal any differences between spatial professionals and controls (*t* = −1.11, df = 25.26, *p* = .28). GLMM cross‐group analyses also do not support differences across groups identified for the relative frequency of use of flat geometric descriptions (*F*(3, 120) = 1.63, *p* = .18), although the distribution (as shown in Fig. 6) is suggestive of a trend that complements the overall patterns of linguistic and conceptual choices. In support of this pattern, within‐group comparisons yielded a highly significant difference between use of flat geometry versus materiality indicators in painters (*F*(1, 120) = 22.36, *p* < .0001), and to a lesser degree in sculptors (*F*(1, 120) = 5.6, *p* < .05) and controls (*F*(1, 120) = 6.93, *p* < .05), but not in architects (*F*(1, 120) = 2.32, *p* = .13)**.**

**Figure 7:**
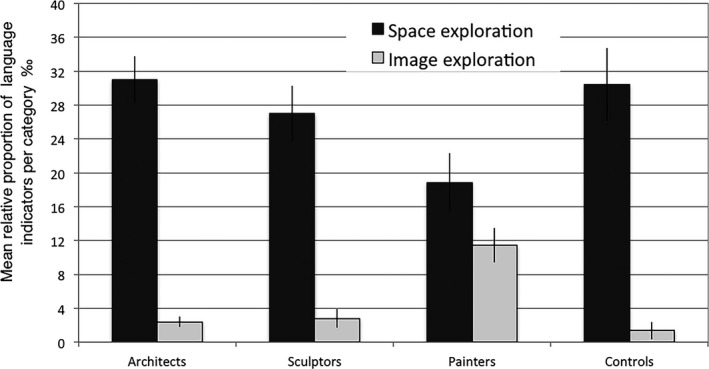
Relative mean proportions (per thousand) and standard errors for the use of linguistic indicators referring to space versus image exploration.

Fig. 7 illustrates the patterns of focus on the exploration of the depicted 3D spaces as opposed to the 2D image itself. Cross‐group variation was also detected in the references to the exploration of the depicted 3D spaces. The results almost reached significance (*F*(3, 120) = 2.6, *p* = .056). However, the groups differed significantly in the relative frequency of language indicators describing a visual exploration of the 2D image (*F*(3, 120) = 12.69, *p* < .0001). The spatial professionals, taken together, differed from controls in their visual exploration (*t* = −2.94, df = 44.24, *p* < .005), but not in their physical (or more embodied) exploration (*t* = 1.021, df = 21.54, *p* = .318) of the depicted environments. Separate between‐group comparisons revealed that this effect was driven by the painters, whose use of language indicators for image exploration was higher than that of controls (*t* = 5.43, df = 120, *p* < .0001), architects (*t* = 4.88, df = 120, *p* < .0001), and sculptors (*t* = 4.66, df = 120, *p* < .0001). No other between‐group comparisons were significant.

**Figure 8:**
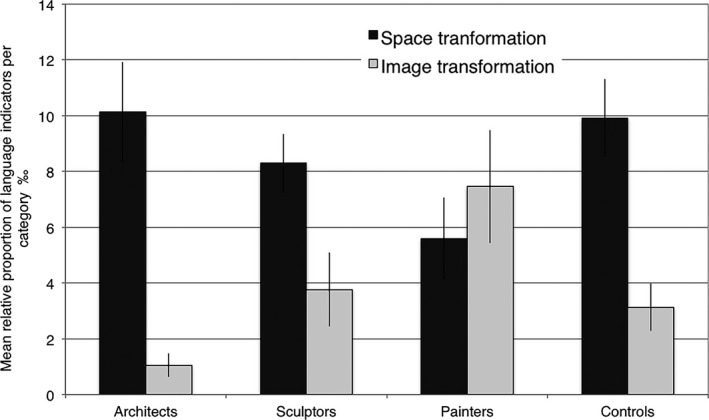
Relative mean proportions (per thousand) and standard errors for the use of linguistic indicators referring to space versus image transformation.

Fig. 8 illustrates the patterns of focus on the transformation of the depicted 3D spaces as opposed to the 2D image itself. This pattern is similar to that shown in Fig. 7, though with lower frequency values. No main effects were found in the relative frequency of use of markers, indicating a transformation of the depicted 3D spaces. A *t* test revealed no main effects between spatial professionals and controls in transforming the 3D spaces (*t* = 0.925, df = 26.88, *p* = .363) or the 2D images (*t* = −0.768, df = 46.72, *p* = .446). However, a GLMM analysis of relative frequency of use of 2D image transformation indicators revealed differences between groups (*F*(3, 120) = 4.23, *p* < .05). This effect was driven by the painters, who used language indicators related to the semantic category of image transformation more frequently than architects (*t* = 3.49, df = 120, *p* < .005).

**Figure 9:**
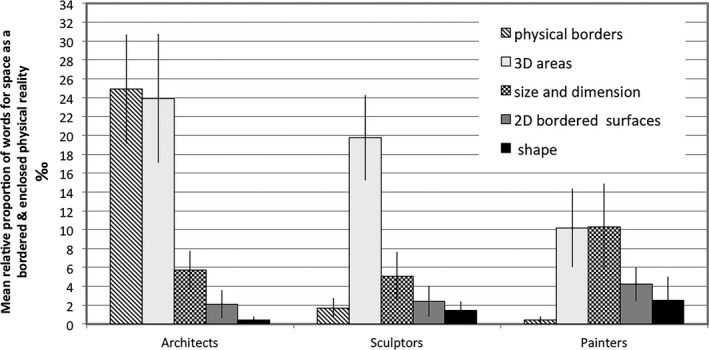
Relative mean proportions (per thousand) and standard errors for the use of linguistic indicators describing space in terms of the subcategories for the notion of space as a bordered physical reality.

Finally, as shown in the previous section, more architects described space as a bordered physical reality than either sculptors or painters. Fig. 9 explores this phenomenon in further detail using the subcategories size/dimension measures, perceivable/physical borders, 3D areas, 2D surfaces, and shape. A cross‐group GLMM analysis of frequency of linguistic indicators belonging to each of these subcategories showed that, specifically, reference to physical borders was significantly different across spatial professional groups (*F*(2, 224) = 10.20, *p* < .0001). This effect was driven by architects who used this category more frequently than either painters (*t* = 4.14, df = 224, *p* < .0001) or sculptors (*t* = 3.63, df = 224, *p* < .005).

## 6 Discussion

Does profession shape how we conceptualize and talk about space? In this study, we addressed this question by analyzing the use of spatial language in a task that does not require specific expertise. We asked to what extent, and in what ways, different conceptions of space find a reflection in natural discourse, beyond the effects of expertise on expert language use within a profession. Our results show a clear and consistent pattern across analysis categories. Painters, sculptors, and architects differed from each other and from a control group in (a) how they treated the images: purely as actual real‐world spaces or both as depicted compositions and real spaces, (b) their focus on the materiality of the environments depicted in the images, and (c) the extent to which they conceived of space in terms of physical borders. Strikingly, these patterns manifested themselves in the groups’ linguistic choices for a particular region (the “furthest point”) in the pictures. However, spatial professionals primarily used either “end” or “back” (depending on profession) in this context, no such pattern could be identified for the control group. Another indication for the different conceptions of space comes from the clarification questions asked; only painters appeared to have difficulty with the notion of “exploring the space” depicted in the images. In the following, we start from a closer look at the two central linguistic indicators “end” and “back,” and then return to the more general patterns of conceptualization.

### 6.1 The case of “end” and “back”

More painters used the term “back” as compared to all other groups, and more architects and painters used the term “end” than controls. What might cause these patterns? We speculate that this may relate to an increased focus in spatial professionals on the dimensionality of the spatial configuration in question and the possible paths through it. In their seminal work, Landau and Jackendoff (1993, p. 221) suggested the following definition for the sides of objects:

If the object is relatively long and narrow, that is, if it has a horizontal generating axis significantly longer than the other axes, it can be said to have *ends*—the regions at the termination of this axis. If the object has a horizontal directed axis, with one that normally faces the observer or determines the normal direction of motion, the region determined by that end of the axis is the object's *front*; the opposite end of this axis determines its *back*.

This indicates a conceptual difference between the two terms, where “end” appears to be more flexible in its use. More precisely, “end” is typically used to refer to one side of an entity as opposed to another side, which is in symmetrical objects equally referred to as “end,” or else distinguished by terms such as “front/rear end” (Talmy, 2000). Crucially, apart from objects, the entity in question could be of a different ontological type, such as “one end of the tunnel” (Talmy, 2000). In the spatial cognition literature, the term is frequently used in relation to directedness and path of motion indicating an “end point,” i.e., destination (Bateman et al., 2010; Talmy, 2000), opposing the source or starting point. In this respect, and related to its literal meaning, the term “end” seems comparable to Allen's (1983) spatial relation “finishes,” which refers to a distance‐related temporal term involving a trail between two points in time and space. Arguably, the term “end” has temporal connotations even in an entirely spatial context such as the one discussed here, indicating a conceptual path through the depicted spaces as if they were real‐world spaces.

In contrast, the semantics of “back,” in its common use, has been defined in relation to the concept of the asymmetric “rear,” as opposed to the “front,” side of an entity (Aurnague & Vieu, 1993; Jackendoff, 1983; Landau, 1991; Talmy, 2000, 2005; Tenbrink, 2011). Furthermore, when describing objects within the visual field, one option is to conceptually divide the observed region into spatial sections and refer to them as “front, back, left, right” (Carroll, 1993; Tenbrink, 2007). This set of terms is also available and frequently used when describing images that show spatial configurations (Gorniak & Roy, 2004; Tenbrink, 2007). Alternatively, a different set of terms can be used that introduces the vertical flat plane, such as “center,” “above,” “below,” “left‐side,” and “right‐side” (Talmy, 2000, 2005). Where “back” is used in 2D contexts, it distinguishes the sagittal from the horizontal direction while neglecting the vertical (Vorwerg, 2009).

Moreover, a prominent notion related to pictorial contexts is that of “background,” as in our excerpt “with what looked like mountains at the back” (Table 2). While the term “background” is arguably more generic and can refer to a spatially extended region, “back” in this case refers only to a specific section of an image (Carroll, 1993), or a subpart of the background. In our study, this specific section was often identified by additional specific details of the image, for example, “at the back where the altar is” or “the back where the boat is in the crevasse.” This meaning, when compared to “end” (as above) seems to reflect a more “static” connotation of the region in question or a fixed location in the picture (Talmy, 2000).

Thus, the architects, sculptors, and painters in our study appeared to conceive of the 3D structure of the space and paths through it, as reflected by their use of “end.” In particular, they consistently used the term to describe a view direction to a specific central area in the pictures on an orthogonal (or *z*) plane moving away from the viewer and toward the center of the picture (if we conceive of the viewer as the origin of the axis forming the planes). This resembles a visual path from the (proximal) outside the (distant) center as if the space depicted represented the real world in three dimensions, rather than from one side to the middle of a 2D picture.

Moreover, the painters differed from the other spatial professionals by also consistently employing a different conceptualization that considers the composition of the image in more static terms, distinguishing functionally and spatially different parts of the picture through the choice of the contrastive term “back.” It is possible that this language pattern in painters is motivated by the basic mechanics of producing paintings, consisting of overlaying static surfaces. Controls, in contrast, did not seem to have any consistent conceptualization to match this pattern.

### 6.2 More general patterns of language use

The distinctive usage pattern of spatial terms to indicate the same region, we argue, is indicative of a more general pattern of conceptualization, where language choices relate systematically to the participants’ professional background. Spatial professionals, and particularly sculptors, referred more frequently to the materiality of the depicted spaces than controls did. Furthermore, painters showed a significant preference toward the use of terms expressing flat geometry instead of materiality more than any of the other groups, with architects not presenting a particular preference between the two.

This pattern was further confirmed when the participants were asked to explore or to transform the space in the images. Painters appeared to have more difficulty with this notion than the other groups, as indicated by the clarification questions they asked. Nevertheless, all groups replied to these prompts by frequently using linguistic indicators pointing to the exploration and transformation of real spaces as depicted in the images, with architects and controls numerically leading, sculptors in an intermediate position, and painters showing least use of 3D space exploration indicators. Crucially, painters additionally made extensive use of language indicators signaling a conceptualization of a transformation of the 2D image itself. Thus, only painters appeared to adopt a dual view that allowed for flexibly switching between two‐ and three‐dimensional conceptualizations. This pattern is also consistent with their propensity to adopt both “end” and “back” in describing the furthest point in the image.

### 6.3 The relation between profession and concepts of space

Further insights about the concepts underlying the distinct linguistic patterns were revealed by the answers given by the spatial professionals when asked about the meaning of “space.” Architects frequently described space in terms of physical boundaries and/or the absence or disappearance of boundaries, which implies taking that feature (the border) as a reference point, “a positive feature must in effect be processed in order to assert its absence” (Rosch & Lloyd, 1978, p. 111). They used language indicators relating to borders, confines, physical limits of containment, and so forth. This is in line with previous literature where architecture is intuitively seen as an “enclosure of spaces” (Behrens, 1910), and space is described as “the void that expands […] between the walls, and is defined by walls” (Endell, 1908; quoted in Forty, 2000), or as a continuum where “boundaries become fluid” (Le Corbusier quoted in Forty, 2000). These concepts were not as prominent in painters and sculptors, who described space in more abstract ways, for example, focusing on the relation between the self/body and the world or outer space.

Taken together, these results support the conclusion that there is a profound link between professional training or daily professional activity based on the constant challenge to modify, represent, design, and create spaces, and a particular type of spatial awareness. This awareness is so deep that it is revealed through systematic conceptual and linguistic differences even in a simple picture description task—a task that, although related to the representation of space, does not require particular expertise in any sense. Indeed, our control groups did not experience any problems with the task—but their linguistic representations were less systematic and, on the whole, less rich than those of spatial professionals.

One interpretation of our results is that professional training and practice in painting, sculpture, and architecture leads to changes in spatial cognition and language. Training and experience would provide a certain *forma mentis* and register (Bhatia, 1993) along with refined verbalization of space (Tenbrink, Coventry, et al., 2011). This interpretation is in line with previous research on expertise‐related cognition (Maguire et al., 2006; Montello, Sullivan, & Pick, 1994; Noordzij et al., 2006; Shipley et al., 2013). On a more specific level, however, our results exceed these previous insights by showing qualitatively distinct patterns of verbalized thought that transcend mere expertise and go beyond the known patterns of using professional jargon. Since the interviews were designed as informal conversations on the simple everyday task of describing and interpreting pictures, they did not require any professional concepts or terminology. Indeed, it would be hard to interpret the patterns of linguistic differences identified in our study in terms of specialized language; the linguistic choices reflected different patterns of concepts, rather than (more predictable) expert jargon.

Another interpretation may be that individual characteristics could lead to a particular choice of professional practice. Indeed, it is quite possible that our choices in life are guided by who we are. In order to pursue this possibility, another type of study is required, for example within‐profession individual longitudinal tests of spatial language and cognitive development.

Our study showed, for the first time, that spatial professionals do not only conceive of space in more refined ways, but arguably adopt a different conception of space that is consistently reflected in meaningful verbalization patterns across various levels of language use. To the extent that language represents thought, then, profession profoundly relates to patterns of thinking. Does this mean that profession actually *shapes* thought, much like culture, with systematic reflections in the use of language (Levinson, 2003; Palmer, 1996)—as Whorf (1941) suggested? Although further research is encouraged to address this question more fully, the evidence provided in this paper indeed represents a step ahead in this direction.

## 7 Conclusion and outlook

Profession profoundly relates to how we think about space. This is reflected systematically in how we talk about spatial environments, even when doing something as simple (and unrelated to profession) as describing a picture. In this study, spatial concepts were related to different professions as follows. Painters focused on flat geometry to a high degree; they conceptualized the depicted spaces in images simultaneously as two‐dimensional pictures and three‐dimensional spaces. Accordingly, they used both “back” (associated with a static, point‐of‐view based conceptualization) and “end” (associated with a dynamic trajectory) for description of a particular region within the pictures. Related to this dual view of depicted space, the notion of “exploring the depicted space” raised questions in this group. Architects focused more on the materiality of the depicted spaces, and easily explored and mentally transformed them consistently as if they were real‐world three‐dimensional spaces. These concepts were reflected by the use of “end” rather than “back.” Sculptors fell in between these two groups; accordingly they used “back” more than architects but less than painters, and “end” more than painters but less than architects. Only their focus on materiality matched that of architects. Non‐spatially‐trained controls focused on flat geometry and explored the depicted spaces similar to architects, but they did not describe the depicted spaces in terms of their materiality, did not explore the spaces in terms of two‐dimensional images, and did not describe, explore, and transform simultaneously in terms of 2D or 3D. Matching these results, almost none of them used the terms “back” and “end.” Although complex in the details, the emerging pattern is clear and consistently highlights a profound relation between profession and spatial concepts that is manifest in various types of linguistic choices.

This insight opens up a broad range of future research avenues, related to the relation between cognition, language, and profession in general, and to the relation between spatial expertise and spatial cognition in particular. Most crucially, related to the Whorfian debate discussed above, the question must be asked to what extent conceptual differences, as related to profession and reflected in language use, affect cognitive processes and reasoning in tasks that are themselves unrelated to profession. If painters verbalize the conceptual task of mentally exploring the space depicted in an image in ways that are fundamentally distinct from other professional groups, what kinds of effects might this have on tasks that involve concrete actions rather than merely verbal description? Does our language and the underlying conceptual patterns, as shaped by our profession, in turn shape our ability and flexibility for tasks that are only remotely or indirectly related to the profession itself? Our research highlights a new way of addressing these questions, namely through the analysis of language use in relation to a conceptually challenging kind of task (cf. Tenbrink, 2015). In future research, a triangulation with relevant performance data should be highly revealing.

Beyond these considerations, the question arises as to where else a relationship between profession and cognition might emerge, if addressed systematically. Although research on individual differences, across fields, increasingly highlights the need for differentiation of distinct populations (Kane & Engle, 2002), most studies still rely on homogeneous groups of participants (typically well‐educated students, mostly studying psychology, of a narrow age range around 20–25 years). However, neuroimaging data point to a general plasticity of the brain in relation to profession (Maguire et al., 2006). If the brain changes with professional experience, this should affect our thinking in rather profound ways. Moreover, a task that triggers fundamentally different conceptualizations in individuals according to their professional background should have distinct repercussions in neuronal activity; this effect still remains to be tested.

Future research will also need to expand the range of verbalizations potentially affected by professional background. While our study focused on picture description only, profession might similarly affect concepts of the real world in everyday settings, for instance during wayfinding, when encountering a new spatially complex environment, when referring to objects in space and their relationships to each other, and other everyday spatial concepts. Enhancing our knowledge about different ways of conceptualizing and talking about space would lead to a better understanding of the challenges involved in communication between different professionals, as seen for instance in the communication between architects, clients, and stakeholders (Tenbrink, Hölscher, Tsigaridi, & Dalton, 2014).

Further research should explore how particular ways of spatial thinking and verbalizing can be proactively exploited and promoted, in order to equip individuals with the skills needed for various activities, professional or other. In this regard, much research is already underway, based on the well‐supported insight that spatial skill supports abstract thinking, with applications in various areas of life (Coleman & Gotch, 1998; Keehner et al., 2004; Peters, Chisholm, & Laeng, 1995; Taylor & Tenbrink, 2013). Our insights may be exploited toward developing specific design principles for purposes of visualization within different domains (Grainger, Mao, & Buytaert, 2016; Skupin & Fabrikant, 2003), taking into account the skills and mindset of diverse stakeholders and professionals.

Finally, the fact that spatial profession is reflected in spatial language use in natural discourse could be exploited as a kind of diagnostic, for instance, in forensic contexts as supporting evidence for the professional background of a speaker or writer (Coulthard, 1994), or for purposes of computational data mining and clustering (Jain, 2010). In such contexts, the prospect of predicting the profession of an individual from the types of linguistic idiosyncrasies that are used in spontaneous discourse could be extremely attractive.

## Supporting information


**Appendix S1:** Experimenter's script: Introduction to the study
**Appendix S2:** PANNS test script
**Appendix S3:** K‐alpha results
**Appendix S4:** GLM analyses (with GLMM comparison).

## Notes

1 In “Sensing Spaces: Architecture Reinvented,” exhibition at Royal Academy, January–March 2014, London.

## Appendix 1 Semantic categories and linguistic indicators

### 1.1

Linguistic indicators were scored independent of the polarity of a statement (positive or negative), since the analysis goal was to identify cognitive focus. Scoring was conservative; if phrases were ambiguous (even under consideration of the context), no scoring was done.

**Table:**

Category	Explanation	Linguistic Indicators Example
Flat geometry	linguistic indicators (e.g., verbs, nouns, adjectives, etc.) suggesting 0‐, 1‐ or 2‐dimensional geometry; linguistic indicators referring to the flat planes of the visual field in a picture	line, lined, square, diagonal, point, triangular, triangle, rectangular, rectangle, edges, plane, planar, to be in line with; background, middle‐ (or center‐)ground, foreground
Materiality	linguistic indicators related to the act of touching or expressing the material or the haptic features (e.g., hardness, weight; material state: liquid, gas, solid; or the make) of an object	touch, push, scratch, kick, punch, solid, solidity, made of ice, icy, made of wood, wooden, woolen, heavy, light, soft, velvet etc
Clarification request	Linguistic expressions containing expressions communicating a possible failure in understanding the task or a doubt. Units containing repetitions that specify a locational doubt. ————— NOT: ‐units in which the apparent doubt is dissipated and answered straightaway by the participants without the prompt of the experimenter (e.g., what do you mean… do you mean… oh, well I would…’) ‐units expressing a general dialogic tendency people have in response to an enquiry by repeating some part (or all) of the question asked (e.g., where would I go?’)	“what do you mean?” “I don't really understand this question” “where would I go … in the image?” “where? In the image or in the space depicted by the image?”
Exploration of the “spaces” in the images	Linguistic expressions (verbal expressions, triggered by the presence of verbs) conveying imagination of navigating or real presence of the body in the spaces depicted through the use of: Verbs of active motion or physical action in space; Verbs of action intended as other ways of exploring an environment; Verbs indicating exploration at a “somatic” level where the agent is a real object that leads the movement of the person within the space depicted.	“I walk up and down” “I jump” “I would talk to the bloke sitting there” “I would make a telephone call …” “the staircase leads me to …” “I'd go where the road takes me …” “where the boat is taking me …”
Exploration of the “images”	Linguistic expressions (verbal expressions, triggered by the presence of verbs) conveying visual/pictorial exploration of the images, such as: Active verbs involving actions to be performed on the picture rather than the space itself, e.g., “drawing” or even verbs of motion; Verbs indicating exploration at a “visual” rather than “somatic” level, where the agent is usually an element of the picture or its geometrical configuration that leads the movement of the person around the picture rather than around the space that is depicted.	“I would draw …” “my eyes want to go” “the diagonal/this 2D image/the picture/the photo is leading my eye to …” “the lines draw me to …”
Transformation of the “spaces” in the images	Linguistic expressions (verbal expressions, triggered by the presence of verbs) conveying active transformation of the spaces as if they were real: Verbs of action that convey change; Verb expressing the intention or the plan to change something in those spaces.	“to put some benches …” “to place some lighting …” “to build some jetties …” “if there was a greater division between the road and the pavement …”
Transformation of the “images”	Linguistic expressions (verbal expressions, triggered by the presence of verbs) conveying transformation or change of the “images” or some aspects of them: Verbs indicating change related to the geometrical configuration of the picture; Verbs indicating changes related to the appearance of the picture.	“to crop people out …” “I would enhance the contrast …” “I zoom in …” “I would mess up with the image …” “I start painting the middle …” “I would straighten up the diagonal …”
Furthest point as “back”	Linguistic indicators (spatial terms) used to identify the furthest point within the picture with the word “back”	“the back there …” “at the back” “towards the back”
Furthest point as “end”	Linguistic indicators (spatial terms) used to identify the furthest point within the image with the word “end”	“the end there” “at the end” “towards the end”
Mental representation of space as a bordered reality (question 4 ONLY)	Linguistic indicators (spatial terms) used to convey the meaning of space as an enclosed and bordered physical reality. We identified specific indicators that pointed to a concept of space in this sense: size and dimension, borders, a 3D defined area, a 2D bordered surface, and the shape of a space	“space is huge” “space is what is enclosed” “the wall” “the ceiling and the floor”
