# Supplementary material for: Sculptors, Architects, and Painters Conceive of Depicted Spaces Differently
Source: Cogn Sci. 2017 Jun 27;42(2):524–53. doi: 10.1111/cogs.12510 (PMC5873447; doi:10.1111/cogs.12510)
Supplement: Supplementary file 1 — Appendix S1: Experimenter's script: Introduction to the study Appendix S2: PANNS test script Appendix S3: K‐alpha results Appendix S4: GLM analyses (with GLMM comparison). [file COGS-42-524-s001.docx]

# Appendix S1

| Hi,  Thanks for your collaboration to this experiment, my name is Claudia Cialone, I am a student at the Psychology and language science department at University College London (UCL) and this study represents my research project supervised by Dr. Hugo Spiers from the Institute of Behavioral Neuroscience.  My interest in this study is to explore how you think about the surrounding space since I understand this being an important variable in your profession.^[[1]](#footnote-1)^  In order to do so I will first ask you some simple questions to test your abstract thinking; I will then show you 6^[[2]](#footnote-2)^ images of places and spaces on paper and I will ask you a few questions in regard so as to have a small talk for each image.  The images are not related in any way.  During the study you will be only voice recorded using an application on my computer and all the information will remain strictly confidential in accordance with the UCL ethical rules and regulations and used anonymously only to draw general conclusions.  During the experiment I cannot answer additional questions you might have, but if you need further clarification about this study you can ask me now. Any questions?  Before we start, I need you to read and fill in this consent form. Also not all the information declared in the consent form will be used since this is just a short experiment and here we are only focusing on cognition.  Just a reminder that at the end of the experiment you will receive a compensation for your participation. |
| --- |

# Appendix S2

# PANNS Test Script:

| This test will be used to analyse your abstract thinking abilities.  **Similarities**  Could you please tell me how are the following pairs of items alike?  Ball and orange  Nickel and dime  Bus and train  The sun and the moon  **Proverbs**  Could you please explain in a few words what the following proverbs mean?  Carrying a chip on your shoulder  Don’t judge a book by its cover  What’s good for the goose is good for the gander  A stitch in time saves nine |
| --- |

# Appendix S3

# K- alpha results

1. K- ALPHA levels of agreement for each scored category in all conditions

| **N** | **Category** | **K-ALPHA** |
| --- | --- | --- |
| 1 | Flat geometry | α =0.92 |
| 2 | Materiality/haptic features | α = 0.86 |
| 3 | Exploration task clarification request | α = 0.84 |
| 4 | Exploration of the ‘spaces’ in the images | α = 0.89 |
| 5 | Exploration of the ‘images’ | α = 0.73 |
| 6 | Transformation of the ‘spaces’ in the images | α = 0.70 |
| 7 | Transformation of the ‘images’ | α = 0.72 |
| 8 | Reference to the frontal point as ‘the back’ | α = 1.00 |
| 9 | Reference to the frontal point as ‘the end’ | α = 1.00 |
| 10 | Mental representation of space as a bordered physical reality | α = 0.72 |

**2. K-ALPHA levels of agreement for each scored subcategory of ‘space as a bordered physical reality’ in all conditions**

| **N** | **Category** | **K-ALPHA** |
| --- | --- | --- |
| 1 | Size and dimension | α = 0.92 |
| 2 | Physical borders | α = 0.94 |
| 3 | 3D enclosed and defined areas | α = 0.81 |
| 4 | 2D bordered surfaces | α = NA* |
| 5 | Shape | α = 1.00 |

***** On this subset of data both the intercorders detected no linguistic indicators to be categorized within the 2D bordered surfaces. So, no variability was detected and no coefficient of disagreement was calculated.

# Appendix S4

# GLM analyses (with GLMM comparison)

The synoptic table below compares the GLMM significant (only) results with the General Linear Model (GLM) results in showing the effects of profession on the frequency of use of cognitively significant semantic categories. In the GLM results the covariates age and years in professional experience (over 6-10 years) are considered into the model. The table shows that even adding the two covariates, the significance of the results (obtained in GLMM) for each category is not affected.

| **Semantic category** | **GLM results** | | **GLMM results** | |
| --- | --- | --- | --- | --- |
|  | **F (3, 60)** | **Sign.** | **F (3, 120)** | **Sign.** |
| Transformation of Space | 0.65 | 0.58 | - | Non sign. |
| Transformation of Image | 3.37 | < 0.05 | 4.23 | < 0.05 |
| Exploration of the Image | 12.18 | < 0.0001 | 12.69 | < 0.0001 |
| Exploration of the Space | 1.15 | 0.336 | - | Non sign. |
| Flat Geometry | 0.87 | 0.46 | - | Non sign. |
| Materiality | 3.45 | 0.02 | 3.52 | < 0.05 |
| Use of ‘back’ | 3.53 | 0.020 | - | Non sign. |
| Use of ‘end’ | 1.81 | 0.15 | - | Non sign. |
| Space defined in terms of physical boundaries | 11.62 | < 0. 0001 | 10.20 | < 0.0001 |

1. the sentence ‘since I understand this being an important variable in your profession’ was only used with architects, painters and sculptors. [↑](#footnote-ref-1)
2. ‘3’ instead of ‘6’ was used with the controls. [↑](#footnote-ref-2)
